# Comparative Analysis of Human Hepatic Lesions in Dengue, Yellow Fever, and Chikungunya: Revisiting Histopathological Changes in the Light of Modern Knowledge of Cell Pathology

**DOI:** 10.3390/pathogens12050680

**Published:** 2023-05-04

**Authors:** Yasmin Pacheco Ribeiro, Luiz Fabio Magno Falcão, Vanessa Cavaleiro Smith, Jorge Rodrigues de Sousa, Carla Pagliari, Edna Cristina Santos Franco, Ana Cecília Ribeiro Cruz, Janniffer Oliveira Chiang, Livia Carício Martins, Juliana Abreu Lima Nunes, Fellipe Souza da Silva Vilacoert, Lais Carneiro dos Santos, Matheus Perini Furlaneto, Hellen Thais Fuzii, Marcos Virgilio Bertonsin Filho, Luccas Delgado da Costa, Maria Irma Seixas Duarte, Ismari Perini Furlaneto, Arnaldo Jorge Martins Filho, Tinara Leila de Souza Aarão, Pedro Fernando da Costa Vasconcelos, Juarez Antônio Simões Quaresma

**Affiliations:** 1 Center for Biological and Health Sciences, State University of Pará, Belém 66087-662, PA, Brazil; 2 Section of Arbovirology and Hemorrhagic Fevers, Evandro Chagas Institute, Ministry of Health, Ananindeua 67030-000, PA, Brazil; 3 School of Medicine, São Paulo University, São Paulo 01246-903, SP, Brazil; 4 Section of Pathology, Evandro Chagas Institute, Ministry of Health, Ananindeua 67030-000, PA, Brazil; 5 Tropical Medicine Center, Federal University of Pará, Belém 66055-240, PA, Brazil; 6 School of Medicine, Federal University of Pará, Altamira 68372-040, PA, Brazil

**Keywords:** arboviruses, histopathology, cell death, necrosis, steatosis, liver

## Abstract

Arboviruses, such as yellow fever virus (YFV), dengue virus (DENV), and chikungunya virus (CHIKV), present wide global dissemination and a pathogenic profile developed in infected individuals, from non-specific clinical conditions to severe forms, characterised by the promotion of significant lesions in different organs of the harbourer, culminating in multiple organ dysfunction. An analytical cross-sectional study was carried out via the histopathological analysis of 70 samples of liver patients, collected between 2000 and 2017, with confirmed laboratory diagnoses, who died due to infection and complications due to yellow fever (YF), dengue fever (DF), and chikungunya fever (CF), to characterise, quantify, and compare the patterns of histopathological alterations in the liver between the samples. Of the histopathological findings in the human liver samples, there was a significant difference between the control and infection groups, with a predominance of alterations in the midzonal area of the three cases analysed. Hepatic involvement in cases of YF showed a greater intensity of histopathological changes. Among the alterations evaluated, cell swelling, microvesicular steatosis, and apoptosis were classified according to the degree of tissue damage from severe to very severe. Pathological abnormalities associated with YFV, DENV, and CHIKV infections showed a predominance of changes in the midzonal area. We also noted that, among the arboviruses studied, liver involvement in cases of YFV infection was more intense.

## 1. Introduction

Arboviruses (arthropod-borne viruses) develop part of their replication cycle in insects and can be transmitted to humans and other animals through the bite of haematophagous arthropods. These viruses have been identified in almost all continents and tend to have a geographic distribution as part of a special ecological subsystem represented by viruses, vectors, amplifying hosts, and reservoirs [1,2,3]. Arboviruses such as yellow fever virus (YFV), dengue virus (DENV), and chikungunya virus (CHIKV), which belong to the *Flavivirus* genus and *Flaviviridae* family (YFV and DENV) and *Alphavirus* genus and *Togaviridae* family (CHIKV), are related to seasonal circulation in tropical and subtropical areas [4,5,6].

These arboviruses develop transmission cycles in wild and urban environments and are transmitted by the bites of mosquitoes belonging to the *Culicidae* family [7,8]. Currently, in Brazil and South America, dengue fever (DF) and chikungunya fever (CF) cases are predominantly urban, whereas the prevalence of yellow fever (YF) is in the sylvatic cycle of the disease, as determined by the epizootics in different regions of the country, mainly in the southeast, northeast, and north [9]. Brazil is considered the main source of the circulation and dispersion of these arboviruses in other Latin American countries. These regions have biotic (forest cover, susceptible hosts, and vectors) and abiotic (temperature, humidity, and rainfall) conditions that determine the diversity, abundance, and spread of vector species populations and influence the dynamics of vector-borne pathogens [10].

YFV, DENV, and CHIKV cause different clinical manifestations in infected individuals and several factors aggravate the disease. They are associated with the characteristics of both the host and viruses, including the patient’s age, viral load, pattern of immune response, secondary infection, and interaction of viral proteins and host cell receptors. This host–virus relationship plays an important role in the clinical outcome of the disease, as it determines not only viral tissue tropism, but also a multitude of intracellular mechanisms that can alter the overall outcome of the infection [11,12,13].

Approximately 15–60% of individuals infected with YFV develop symptoms of the disease, with death occurring in 20–50% of cases [14,15]. In DF cases, lethality is also associated with the infecting serotype (DENV-1 to DENV-4), which influences patient clinical outcomes [16]. Most infected individuals with CF develop mild symptoms. Severe cases tended to have atypical disease characteristics (hepatitis, myocarditis, and neuropathies) [17,18].

These arboviruses have been observed to replicate and cause significant damage to various organs of the host, such as the heart, lung, thymus, spleen, kidney, and liver; therefore, they are considered multi-organic pathogens [18,19,20,21,22]. The liver is considered a target organ for YFV and DENV infections and is characterised by CHIKV infection [23,24]. The pathogenesis of liver injury involves several mechanisms including direct viral cytopathic effects and immune-mediated injury [25].

Patients infected with these viruses can be diagnosed using several techniques, including serology, viral isolation, immunohistochemistry (IHC), molecular biology, and histopathology [26,27,28,29,30,31,32,33]. Previous studies in animal models and histopathological results in humans have allowed the characterisation of the clinical and pathological changes that occur during viral pathogenesis [34]. The hepatic histopathological patterns observed in the pathogenic profiles of these arboviruses show different degrees of degenerative lesions, with the presence of inflammatory infiltrate, macro- and microvesicular steatosis, necrosis, alterations in hepatic sinusoidal endothelial cells (HSEC), and Kupffer cells [24,35,36,37,38,39,40,41,42,43,44,45,46,47,48,49,50,51,52,53,54,55,56,57,58].

Arbovirus infections are deadly in several areas of the world. However, little is known about the involvement of the target compartments in fatal arboviral diseases. In addition, these infections induce tissue damage at the cellular level; however, their roles in the pathogenesis are poorly understood. In this study, we aim to characterise the histopathological features of the liver compartments in infections induced by two viruses of the *Flaviviridae* family (YFV and DENV) and one of the *Togaviridae* family (CHIKV).

## 2. Materials and Methods

This is an analytical cross-sectional study using retrospective samples from patients with confirmed laboratory diagnoses who died due to infection and complications of YFV, DENV, and CHIKV. The patients’ ages ranged from 15 to 63 years (mean age = 37 years), and 60% were male. The samples came from the states of Tocantins, Goiás, Distrito Federal, and Paraíba, where most cases of infections came from the state of Goiás, between 2000 and 2016.

Samples collected from patients with a confirmed diagnosis of YFV, DENV, or CHIKV infection based on clinical/epidemiological, histopathological, IHC, and molecular criteria were included.

Samples without confirmatory histopathological, IHC, or molecular reports were excluded. Samples that did not have enough material to make slides, that is, the amount of material was not enough to make slides, and their use could compromise the storage of a part for return to the laboratory block file were also excluded. In addition, the identified samples were impaired.

A total of 70 liver tissue samples from individuals (49 men and 21 woman) with YF, DF, and CF (36 samples of YF, 17 of DF, and 17 of CF) were obtained from the biobank of the Pathology Section of the Evandro Chagas Institute (SAPAT/IEC), located in the Municipality of Ananindeua, collected via epidemiological surveillance between 2000 and 2017, were included in this study.

Liver samples obtained during routine autopsies were used as negative controls. Twenty liver samples were obtained from patients with a negative molecular diagnosis for the main hepatotropic viruses, with no morphological changes, from the Death Verification Service of the Metropolitan Region of the city of Belém, State do Pará, Brazil (Supplementary Figure S1).

All procedures performed in this study were approved by the Research Ethics Committee of the Evandro Chagas Institute, following the recommendations of the National Health Council No. 466/12. This was approved by the perish number 2.364.226. The requirement for free and informed consent was waived, as the samples collected were obtained post-mortem, and all data were handled and analysed anonymously, without the nominal Identification of individuals.

### 2.1. Histological Analysis

Liver tissue samples were fixed for 24 h in a 10% buffered formalin solution and stored in ethyl alcohol. Tissue fragments were passed through several increasing alcohol solutions (70–100%). Two passages were carried out in xylene and two were carried out in paraffin at 60 °C. After cooling, the tissue blocks were sectioned using a rotating microtome to obtain 5 µm thick sections. Tissue sections were stained with haematoxylin and eosin (H&E).

To evaluate the histopathological patterns, the stained samples were visualised using a microscope at a total magnification of 400× in the area or zone of the hepatic acinus of Rappaport (Z1 = periportal, Z2 = midzonal, and Z3 = centrilobular) and the portal tract (PT). Damage was measured according to a semiquantitative scale from 0 to 4 (0: absent, 1: mild +, 2: moderate ++, 3: intense +++, 4: very intense ++++) to classify the degree of tissue damage observed, as described by Quaresma et al. [35].

Morphological alterations in the hepatic acinus (Z1, Z2, and Z3), PT, and inflammatory infiltrate included cell swelling, macrovesicular steatosis, microvesicular steatosis, lytic necrosis, coagulative necrosis, apoptosis (Councilman–Rocha Lima body), hyperplasia of Kupffer cells, Kupffer cell hypertrophy, sinusoidal endothelial alteration (SEA), sinusoidal congestion, sinusoidal dilatation, edema, congestion, portal vein alteration, portal artery alteration, biliary canaliculus alteration, and lymphocytes, neutrophils, plasma cells, and macrophages.

### 2.2. Immunohistochemistry

Tissue immunostaining was performed using hyperimmune mouse ascitic fluids for YFV, DENV, and CHIKV produced in young mice that were titrated to select the best dilution for the immunohistochemical assay [58]. Initially, the tissue samples were deparaffinised in xylene and hydrated in a series of decreasing concentrations of ethyl alcohol (90%, 80%, and 70%). Subsequently, antigenic recovery was performed in protease solution (Sigma-Aldrich, Poole, UK) at 37 °C. Non-specific proteins were blocked with a solution of 5% skim milk, 0.01 M phosphate-buffered saline (PBS), and normal horse serum (Vector Laboratories, Burlingame, CA, USA). Anti-YFV, anti-DENV, and anti-CHIKV antibodies diluted 1:100 in PBS pH 7.2 were added to the slides and incubated at room temperature for 1 h. After 3 washes with PBS, it was treated with a biotinylated anti-mouse IgG secondary antibody (Vector Laboratories, Burlingame, CA, USA) diluted 1:5 and incubated for 1 h, followed by 3 washes with PBS. Next, the sections were incubated with streptavidin–alkaline phosphatase complex (Invitrogen Corporation, Camarillo, CA, USA) 1:20 dilution for 1 h at room temperature, after which the slides were washed 3 times with 0.1 M TRIS solution. The HistoMark Red Phosphatase Kit (KPL, Gaithersburg, MD, USA) was applied to the slides for 30 min at room temperature for the detection of the antibody staining. The sections were then counterstained with Harris haematoxylin and the slides were assembled with Entellan.

### 2.3. Molecular Biology

All samples used in the present study were submitted to refrigeration, followed by freezing at −20 °C, −70 °C, or immersed in liquid nitrogen. Biological samples of human blood and tissue were subjected to diagnostic tests for YFV, DENV, and CHIKV using real-time polymerase chain reaction (PCR), according to the protocol described by Mori et al. [30].

### 2.4. Statistical Analysis

Data were analysed using descriptive and inferential statistics according to the nature of the variables, summarised by measures of central tendency and dispersion, in addition to the confidence interval. This was expressed in the form of graphics. To compare the different groups in terms of morphological changes, inflammatory infiltrate, and SEA in each acinar zone, in addition to structural changes and inflammatory infiltrate observed in the PT, the Kruskal–Wallis analysis of variance was used, followed by Dunn’s post hoc test.

In comparisons of the same group in relation to a given variable but in different zones, the comparison was made using Friedman’s analysis of variance with Dunn’s post hoc test. The results obtained were stored in Microsoft Excel 2019 spreadsheets, and graphs were constructed using GraphPad Prism 9.2 (GraphPad Software, San Diego, CA, USA). Differences were considered statistically significant at *p* ≤ 0.05.

## 3. Results

### 3.1. Qualitative Histology

The qualitative analysis of the histopathological aspects showed that cell swelling, steatosis, and apoptosis were the most frequent hepatic alterations observed in the YF, DF, and CF samples in all areas of the hepatic acinus. However, prevalence was observed in the midzonal area. Histopathological changes and viral antigen immunostaining were absent in the normal control samples (Supplemental Figure S1).

**Figure 1:**
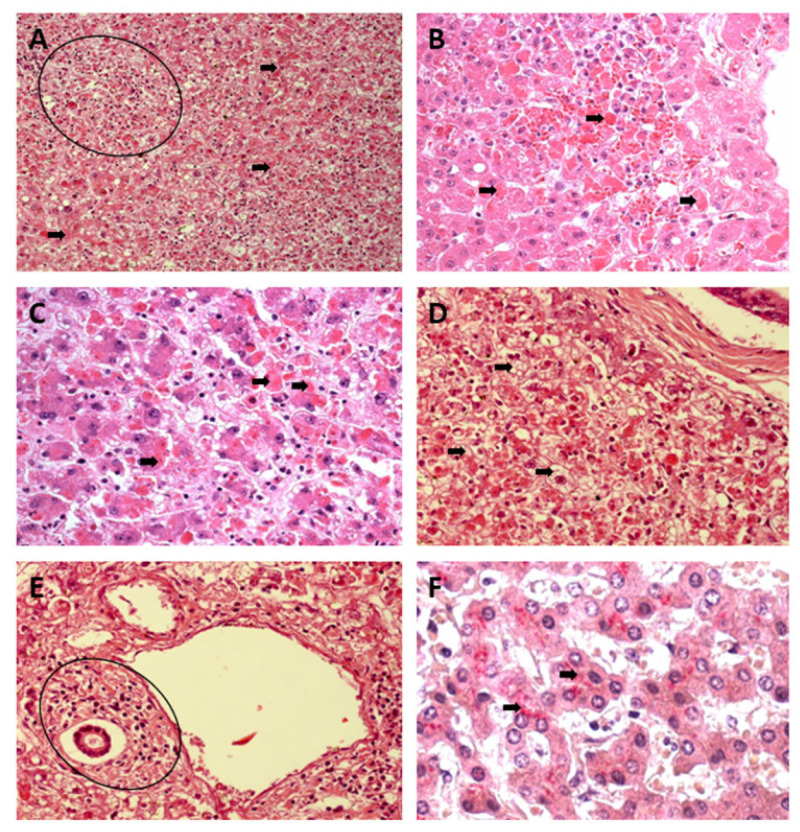
Histopathology of hepatic infection caused by YFV. The presence of multiple apoptotic bodies (black arrows) and mononuclear acinar inflammatory infiltrate are observed (circle) (**A**). In (**B**), apoptotic bodies (Councilman bodies) and haemorrhage are observed (black arrows). In (**C**) and (**D**), multiple apoptotic and Councilman bodies and macro- and microvesicular steatosis are observed (black arrows). In (**E**), portal tracts with inflammatory infiltrate of mononuclear cells (circle) are observed. In (**F**), the presence of antigens for YFV in liver tissue marked by IHC are observed (black arrows). (Hematoxylin and Eosin, (**A**,**E**): 200×; (**B**–**D**,**F**): 400×). YFV, yellow fever virus; IHC, immunohistochemistry.

Councilman’s acidophilic bodies, representing hepatocytes in an apoptotic process, demonstrated by a homogeneous, intensely acidophilic mass, were frequently observed in the three zones of the YF and DF samples and were absent in the CF cases. However, the process showed greater comparative intensity in the midzonal area of the YF cases (Figure 1A–F).

**Figure 2:**
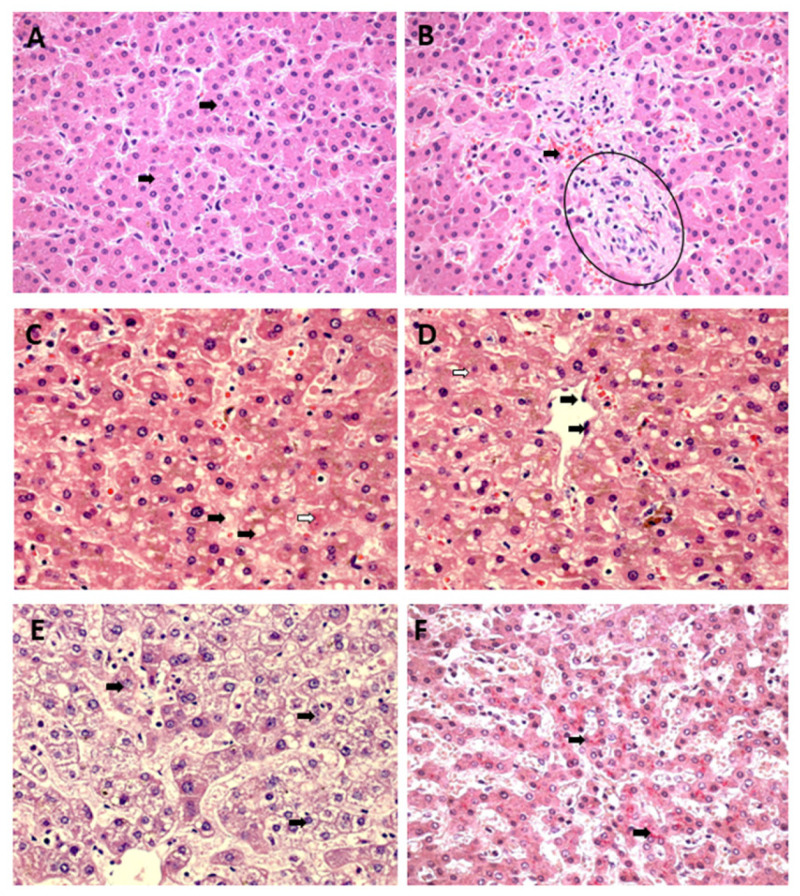
Histopathology of hepatic infection caused by DENV. The presence of mild cell swelling (black arrows) is observed in (**A**) associated with a mononuclear inflammatory infiltrate (circle) in (**B**) and the presence of macrovesicular steatosis (black arrows) and apoptosis patterns (white arrows) in (**C**,**D**). Changes in endothelial cells associated with the inflammatory response of mononuclear cells (black arrows) are observed in (**D**). Hepatocytic ballooning and lytic necrosis (black arrows) of hepatocytes are observed in (**E**) and immunostaining by IHC by alkaline phosphatase (black arrows) in liver tissue is observed in (**F**). (Hematoxylin and Eosin, (**A**,**B**): 200×; (**C**–**F**): 400×). DENV, dengue virus; IHC, immunohistochemistry.

Small focal points of lytic and coagulative necrosis were also observed in the hepatocytes of the YF, DF, and CF cases, which were generally mild in the three analysed zones. However, when lytic necrosis was observed in Z2 of patients with YF, DF, and CF, a predominance of this alteration was observed in the area. These focal points of necrosis were visualised as areas of parenchyma showing a complete absence of cells and were generally replaced by an amorphous and slightly eosinophilic mass containing cellular debris (Figure 2A–F).

**Figure 3:**
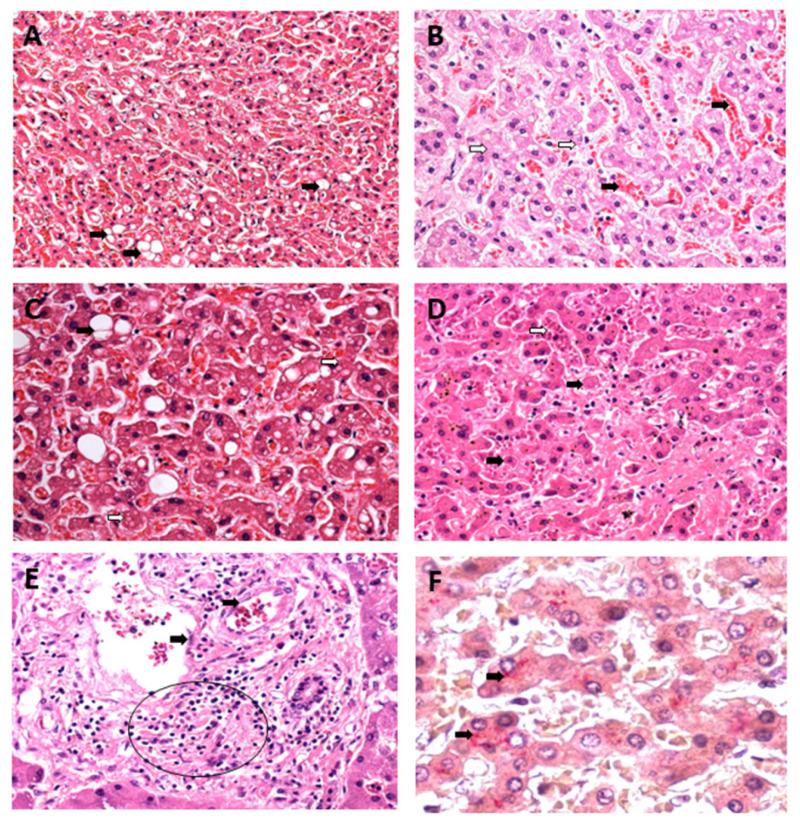
Histopathology of hepatic infection caused by CHIKV. The presence of macrovesicular steatosis (black arrows) (**A**) in the midst of sinusoidal congestion (white arrows) and cell swelling (white arrows) is observed in (**B**), and the presence of macro (black arrows) and microdroplet (white arrows) steatosis patterns of steatosis is observed in (**C**). Figures of cells with morphology in apoptosis (black arrows) and necrosis (white arrows) is observed in (**D**), associated with the response mononuclear inflammatory disease (black arrows and circle) in (**E**) and the presence of immunostaining for the virus in tissue by IHC by alkaline phosphatase (black arrows) in (**F**). (Hematoxylin and Eosin, (**A**): 200×; (**B**–**F**): 400×). CHIKV, chikungunya virus; IHC, immunohistochemistry.

Mild to moderate hyperplasia and hypertrophy of Kupffer cells were other alterations observed in the acinar zones (Z1, Z2, and Z3), with an emphasis on the higher frequency of Z2 alterations in all cases studied. In addition, fatty changes characterised by macro- and microvesicular steatosis in the cytoplasm of hepatocytes were accompanied by mild-to-moderate acinar and portal inflammatory infiltrates consisting of lymphocytes, plasma cells, neutrophils, and macrophages, with a higher frequency of *lymphoplasmacytic* infiltrates, especially around the foci of necrosis. The distribution of inflammation in the lobule maintained a pattern similar to that of other acinar changes, with a clear preference for the midzonal area (Figure 3A–F).

Among the endothelial alterations observed in the samples, there was a difference in the histopathological findings between the arboviruses studied; in the SEA cases, YF and CF showed a higher frequency of this alteration. Regarding sinusoidal congestion, the YF and DF cases stood out and were more intense. Sinusoidal dilatation was highlighted for the CF cases, with no change in the YF and DF cases. A predominance of endothelial alterations was observed at Z2.

In the PT, the main alterations analysed were moderate, with oedema equivalent to the three arboviruses studied; in portal vein alteration, the DF cases were more intense. Congestion and alteration of the biliary canaliculus were mild and observed only in the patients with YF and DF. However, the alteration of the portal artery was mild in all three arboviruses studied.

### 3.2. Semiquantitative Histology

#### Histopathological Changes in Hepatic Acinus

**Figure 4:**
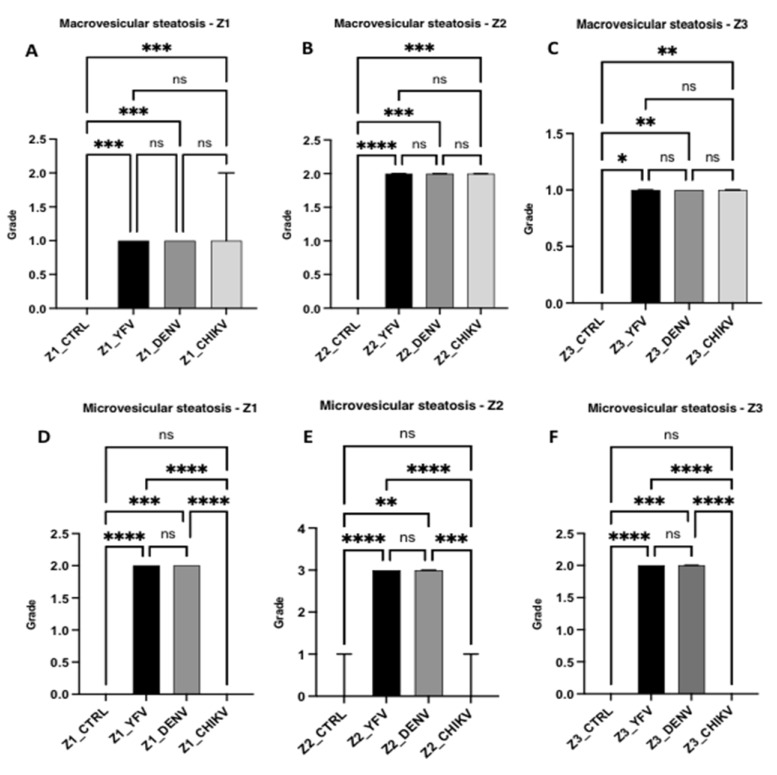
Semiquantification of histopathological changes in hepatic acini (Z1, Z2, and Z3) of patients infected with YFV, DENV, and CHIKV. Macrovesicular steatosis (**A**–**C**) and microvesicular steatosis (**D**–**F**). ns: *p* > 0.05, * *p* ≤ 0.05, ** *p* ≤ 0.01, *** *p* ≤ 0.001, **** *p* ≤ 0.0001. YFV, yellow fever virus; DENV, dengue virus; CHIKV, chikungunya virus.

**Figure 5:**
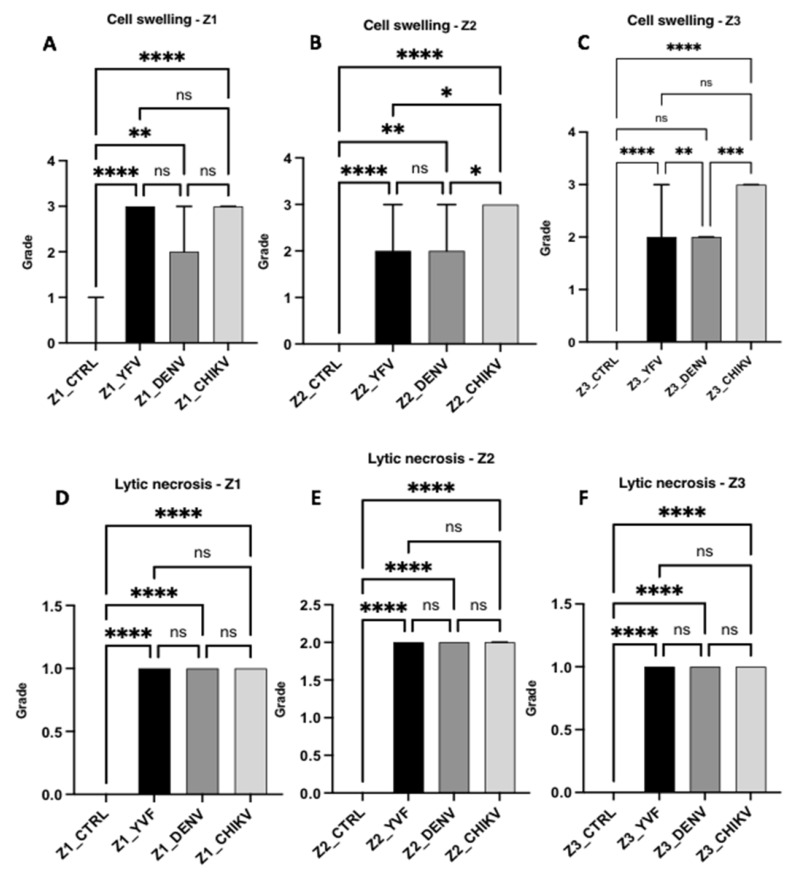
Semiquantification of histopathological changes in hepatic acini (Z1, Z2, and Z3) of patients infected with YFV, DENV, and CHIKV. Cell swelling (**A**–**C**) and lytic necrosis (**D**–**F**). ns: *p* > 0.05, * *p* ≤ 0.05, ** *p* ≤ 0.01, *** *p* ≤ 0.001, **** *p* ≤ 0.0001. YFV, yellow fever virus; DENV, dengue virus; CHIKV, chikungunya virus.

The semiquantitative data on histopathological changes in hepatic acinus showed a significant difference between the control and analysed variables in all zones of the hepatic acinus. Intense cellular swelling was observed in the three hepatic zones of the CF cases. In the case of YF, only Z1 showed a high swelling intensity, whereas the other two zones showed a moderate intensity. In the DF cases, the swelling was moderate in the three analysed zones. In the individual analyses, the DF and CF cases presented homogeneous frequency and intensity between the hepatic acinus zones for this alteration (Figure 4 and Figure 5).

Regarding the fatty alterations characterised by macrovesicular and microvesicular steatosis, in macrovesicular steatosis, the results were similar between the arboviruses and the specific studied zones, with no significant differences between the YF, DF, and CF cases; however, there was a significant difference between the control and the cases (Z1 and Z2: ctrl × yfv, *p* < 0.001/ctrl × denv, *p* < 0.001/ctrl × chikv, *p* < 0.001; Z3: ctrl × yfv, *p* < 0.05/ctrl × denv, *p* < 0.001/ctrl × chikv, *p* < 0.001). However, when the zones were separately analysed in each case, the intensity of the alteration was mild in Z1 and Z3, whereas in Z2 of all arboviruses, an increase in the quantification of the alteration was observed, which was moderate in all cases studied. Thus, the macrovesicular steatosis identified in the midzonal area of the YF, DF, and CF cases seemed to show higher values than those in other areas of the hepatic acinus (Figure 4A–C).

In microvesicular steatosis, there was a clear difference between the arboviruses from the *Flaviviridae* and *Togaviridae* families (Z1, Z2, and Z3: yfv × chikv, *p* < 0.0001/denv × chikv, *p* < 0.0010; Z1 and Z3: yvf × denv, *p* > 0.9999; Z2: yfv × denv, *p* = 0.3008); in Z1 and Z3, the YF and DF cases presented moderate intensity, and in Z2, the change was intense for both infections. However, this alteration was absent in the CF cases (Figure 4D–F).

**Figure 6:**
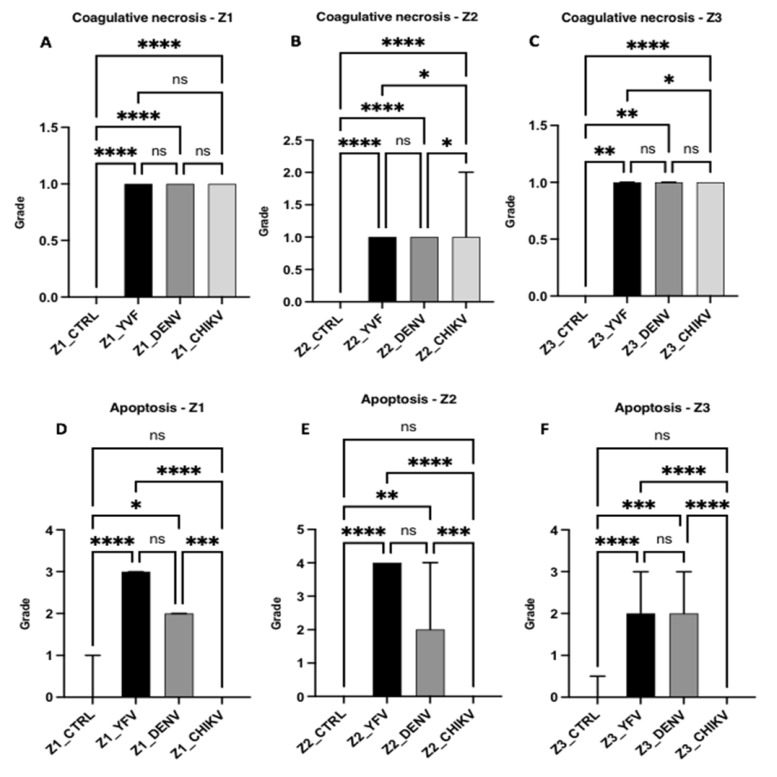
Semiquantification of histopathological changes in hepatic acini (Z1, Z2, and Z3) of patients infected with YFV, DENV, and CHIKV. Coagulative necrosis (**A**–**C**) and apoptosis (**D**–**F**). ns: *p* > 0.05, * *p* ≤ 0.05, ** *p* ≤ 0.01, *** *p* ≤ 0.001, **** *p* ≤ 0.0001. YFV, yellow fever virus; DENV, dengue virus; CHIKV, chikungunya virus.

Regarding the necrotic processes evaluated, in relation to lytic necrosis, the liver samples from the patients with YF, DF, and CF showed no significant difference when compared to each other (Z1 and Z3: yfv × denv, *p* > 0.9999/yfv × chikv, *p* > 0.9999/denv × chikv, *p* > 0.9999; Z2: yfv × denv, *p* > 0.9999/yfv × chikv, *p* = 0.1033/denv × chikv, *p* = 0.2460); however, there was a significant difference between the control and infection groups (all zones: ctrl × yfv, *p* < 0.0001/ctrl × denv, *p* < 0.0001/ctrl × chikv, *p* < 0.0001). Although the values for lytic necrosis were equivalent between the arboviruses, necrosis was mild in Z1 and Z3 and moderate in Z2 (Figure 5). Regarding coagulative necrosis, all areas of the three infections corresponded to a mild degree (Figure 6), with different results between the patients with YF, DF, and CF in Z2 (yfv × denv, *p* > 0.9999/yfv × chikv, *p* = 0.0185/denv × chikv, *p* = 0.0239) and Z3 (yfv × denv, *p* > 0.9999/yfv × chikv, *p* = 0.0259/denv × chikv, *p* = 0.1594), but without significant differences in Z1 (yfv × denv, *p* > 0.9999/yfv × chikv, *p* = 0.9011/denv × chikv, *p* > 0.9999) (Figure 6).

Apoptosis was absent in all the analysed zones in all the CF cases when compared with the controls (*p* > 0.9999). In the DF cases, there was a significant difference between the control and infection groups (Z1: ctrl × denv, *p* = 0.0161; Z2: ctrl × denv, *p* = 0.0064; Z3: ctrl × denv, *p* = 0.0008), with moderate apoptosis observed in all zones. The YF cases were related to the highest values of apoptotic processes of the arboviruses studied, emphasising the midzonal area and presenting a very intense lesion (all zones: ctrl × yfv, *p* < 0.0001). The periportal and centrilobular areas of the YF samples showed intense and moderate apoptosis, respectively (Figure 6).

The other changes analysed included Kupffer cell hyperplasia and hypertrophy. Regarding hyperplasia, the DF cases differed significantly between the control and infection groups in all evaluated areas (Z1: ctrl × denv, *p* = 0.0013; Z2: ctrl × denv, *p* < 0.0001; Z3: ctrl × denv, *p* = 0.0435). However, the YF and CF cases showed no change in Z3 (ctrl × yfv, *p* = 0.1696/ctrl × chikv: *p* > 0.9999/yfv × chikv: *p* > 0.9999). Z1 and Z2 showed similar lesion intensities with mild and moderate hyperplasia, respectively, for all three arboviruses.

**Figure 7:**
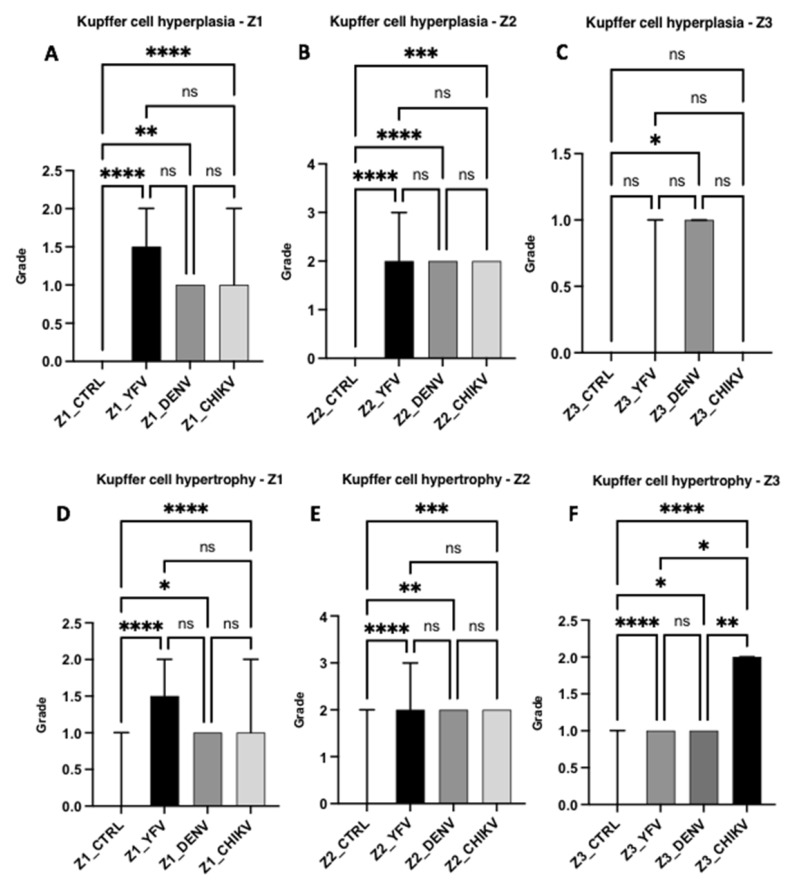
Semiquantification of histopathological changes in hepatic acini (Z1, Z2, and Z3) of patients infected with YFV, DENV, and CHIKV. Kupffer cell hyperplasia (**A**–**C**) and Kupffer cell hypertrophy (**D**–**F**). ns: *p* > 0.05, * *p* ≤ 0.05, ** *p* ≤ 0.01, *** *p* ≤ 0.001, **** *p* ≤ 0.0001. YFV, yellow fever virus; DENV, dengue virus; CHIKV, chikungunya virus.

Regarding Kupffer cell hypertrophy, all arboviruses showed a significant difference between the control and infection groups in the different hepatic zones. All the arboviruses showed mild and moderate hypertrophy for Z1 and Z2, respectively (Z1: ctrl × yfv, *p* < 0.0001/ctrl × denv, *p* = 0.0149/ctrl × chikv, *p* < 0.0001; Z2: ctrl × yfv, *p* < 0.0001/ctrl × denv, *p* = 0.0017/ctrl × chikv, *p* = 0.0008). In Z3, however, there was a difference between the arboviruses; the CF cases showed moderate cellular hypertrophy, while the YF and DF cases showed mild hypertrophy (yfv × chikv, *p* = 0.0192/denv × chikv, *p* = 0.0020/ctrl × yfv, *p* < 0.0001/ctrl × denv, *p* = 0.0104/ctrl × chikv, *p* < 0.0001). Regarding hypertrophy, the CF cases showed a higher frequency of moderate intensity than the YF and DF cases. Among the evaluated zones, the overall midzonal area showed a greater commitment. However, in the CF cases, the midzonal and centrilobular areas were equally affected (Figure 7).

**Figure 8:**
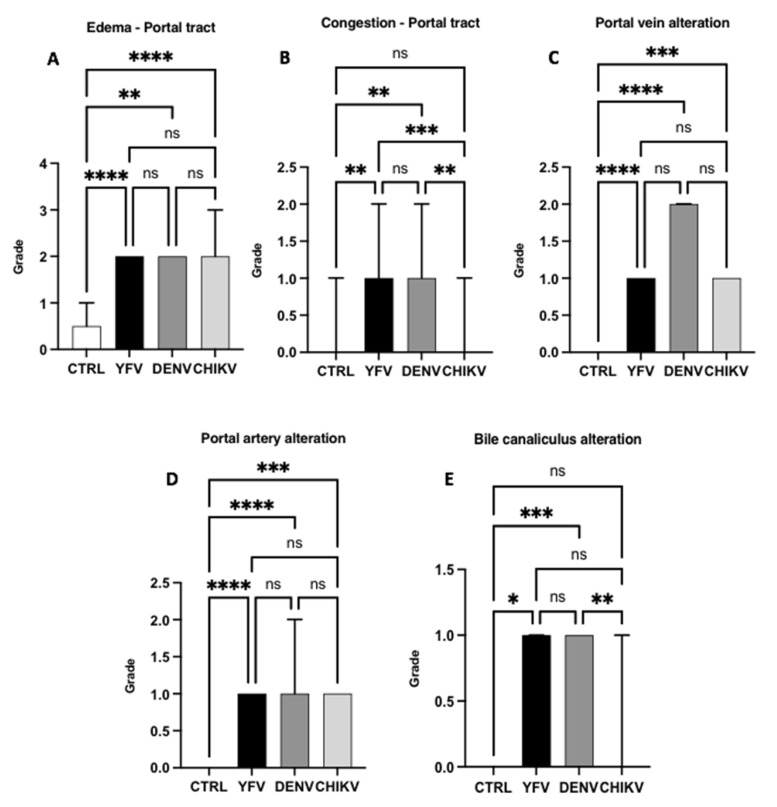
Semiquantification of histopathological changes in the portal tract of patients infected with YFV, DENV, and CHIKV. Edema (**A**), congestion (**B**), portal vein alteration (**C**), portal artery alteration (**D**), bile canaliculus alteration (**E**). ns: *p* > 0.05, * *p* ≤ 0.05, ** *p* ≤ 0.01, *** *p* ≤ 0.001, **** *p* ≤ 0.0001. YFV, yellow fever virus; DENV, dengue virus; CHIKV, chikungunya virus.

Regarding the semiquantification of endothelial changes in the hepatic acinus of the YF, DF, and CF cases, three variables, including SEA, were significantly different between the control and infection groups. In all three evaluated areas, the YF and CF cases expressed a moderate SEA profile (Z1 and Z2: yfv × chikv, *p* > 0.9999/Z3: yfv × chikv, *p* = 0.2708), whereas only Z2 showed moderate injury in the DF cases (yfv × denv: *p* = 0.7662/denv × chikv: *p* > 0.9999), and in Z1 and Z3, the SEA was light (Z1: yfv × denv, *p* = 0.2558/denv × chikv, *p* = 0.0454; Z3: yfv × denv, *p* = 0.3483/denv × chikv, *p* = 0.0049). Among the arboviruses studied, SEA was predominant in the YF and CF cases; in all infections, Z2 was the most affected (Figure 8).

The second variable analysed was sinusoidal congestion. It was absent in the CF cases in all analysed zones (Z1: ctrl × chikv, *p* = 0.3037; Z2: ctrl × chikv, *p* = 0.7697; Z3: ctrl × chikv, *p* = 0.4282). Significant values were referred to cases of YF and DF (Z1: ctrl × yfv and ctrl × denv, *p* = 0.0029; Z2: ctrl × yfv, *p* = 0.0012/ctrl × denv, *p* = 0.0349; Z3: ctrl × yfv, *p* = 0.0200/ctrl × denv, *p* = 0.1226), with corresponding results in Z1 and Z3, with mild degree (Z1 and Z3: yfv × denv, *p* > 0.9999). However, sinusoidal congestion in Z2 for the YF cases was moderate, whereas in the DF cases, the same area presented with mild congestion (yfv × denv: *p* > 0.9999). Sinusoidal congestion was prevalent in the YF and DF cases, with greater intensity in Z2 of the YF cases.

Sinusoidal dilatation is the final alteration associated with the hepatic endothelium. This alteration was absent in the YF and DF cases in all analysed zones (all zones: ctrl × yfv, ctrl × denv, and yfv × denv, *p* > 0.9999). However, in the CF cases, dilation was moderate and homogeneous in all three studied areas (all zones: ctrl × chikv, yfv × chikv, and denv × chikv, *p* < 0.0001). We observed a clear predominance of sinusoidal dilatation in the CF cases compared to the YF and DF cases, without a specific zone of involvement.

Among the changes observed in the PT, oedema was moderate in the YF, DF, and CF cases (ctrl × yfv: *p* < 0.0001/ctrl × denv: *p* = 0.0014/ctrl × chikv: *p* < 0.0001), and there was no significant difference between the arboviruses (yfv × denv: *p* = 0.8430/yfv × chikv: *p* > 0.9999/denv × chikv: *p* > 0.9999) (Figure 6A). In the case of portal congestion, the YF and DF cases were mild (yfv × chikv: *p* = 0.0008/denv × chikv: *p* = 0.0053/yfv × denv: *p* > 0.9999), and in the CF cases, changes were absent (ctrl × chikv: *p* > 0.9999) (Figure 8). Regarding the alteration of the portal vein, the DF cases presented higher values than the YF and CF cases, although it was not statistically significant (yfv × denv: *p* > 0.9999/denv × chikv: *p* = 0.2064), and it was moderate in the DF cases and light in the YF and CF cases (Figure 8).

There was no significant difference in portal artery alterations between the arboviruses studied (yfv × denv: *p* > 0.9999/yfv × chikv: *p* > 0.9999/denv × chikv: *p* = 0.3789), presenting a mild degree of alteration only between the control and samples. However, when the bile canaliculus alteration was evaluated, a predominance in the YF and DF cases was observed (ctrl × yfv: *p* = 0.0201/ctrl × denv: *p* = 0.0001/yfv × denv: *p* = 0.1159) with a mild degree of alterations, whereas there was no change in the CF cases (ctrl × chikv: *p* > 0.9999/yfv × chikv: *p* = 0.3598)/denv × chikv: *p* = 0.0018) (Figure 8).

The acinar inflammatory infiltrate was mild to moderate among the arboviruses studied, with a significant difference between the control and infection groups. Of the cell types of inflammatory cells, lymphocytes in Z1 and Z3 of the YF, DF, and CF cases presented mild intensity, with no significant difference between the inflammatory processes of the infections evaluated in these areas of the hepatic acinus (Z1 and Z3: yfv × denv, *p* > 0.9999/yfv × chikv, *p* > 0.9999/denv × chikv, *p* > 0.9999), Z2 of the YF and DF cases had higher values when compared to Z2 of the CF cases, with moderate and light infiltration, respectively (yfv × denv: *p* > 0.9999/yfv × chikv: *p* < 0.0001/denv × chikv: *p* = 0.0015).

The plasma cell infiltrates identified in the hepatic acinus of the fatal cases of YF, DF, and CF were equivalent to the lymphocytic infiltrates in Z1 and Z3 of the cases studied, with mild intensity and no significant difference between the arboviruses (Z1 and Z3: yfv × denv, *p* > 0.9999/yfv × chikv, *p* > 0.9999/denv × chikv, *p* > 0.9999). However, unlike the lymphocytic infiltrate, the evaluation of plasma cell infiltrate in Z2 of the arboviruses showed an increase only in the YF cases with a moderate degree of intensity (yfv × denv: *p* = 0.5215)/yfv × chikv: *p* = 0.0003/denv × chikv: *p* = 0.2519).

Neutrophil acinar inflammatory infiltrate was not identified in Z1 and Z3 of the CF cases (Z1: ctrl × chikv, *p* > 0.9999/yfv × chikv, *p* = 0.1939/denv × chikv, *p* = 0.1023; Z3: ctrl × chikv, *p* = 0.3206/yfv × chikv, *p* > 0.9999/denv × chikv, *p* > 0.9999). In the YF cases, the Z1 and Z2 zones presented a mild intensity. Neutrophil infiltration was absent in Z3 of the YF cases. In the DF cases, the infiltrate was homogeneous between the analysed zones, all of which had a mild intensity. Preferential occurrence of acinar inflammatory infiltrate in the middle zone of the hepatic acinus was observed. Among the arboviruses studied, the YF and DF infection cases showed a greater intensity of inflammatory cells in the liver tissue than the CF cases.

Regarding the portal inflammatory infiltrate, the lymphocytic inflammatory infiltrate was moderate in the YF and DF cases and mild in the CF cases (yfv × denv: *p* = 0.1139/yfv × chikv: *p* = 0.0073/denv × chikv: *p* > 0.9999). Similar to the acinar infiltrate values, the plasma cell infiltrates in the YF cases were comparatively higher than those in the DF and CF cases, corresponding to moderate and mild degrees, respectively, although the difference was not statistically significant (yfv × denv: *p* = 0.8623/yfv × chikv: *p* > 0.9999/denv × chikv: *p* > 0.9999). In the PT, no neutrophil inflammatory infiltrate was identified in the CF cases (ctrl × chikv: *p* > 0.9999/yfv × chikv: *p* = 0.0012/denv × chikv: *p* = 0.0053), and only mild infiltrate was identified in the YF and DF cases (yfv × denv: *p* > 0.9999). The last inflammatory cell specimens identified in PT were macrophages, and no significant differences were observed between the YF, DF, and CF samples, which showed light macrophage infiltration (yfv × denv: *p* > 0.9999/yfv × chikv: *p* > 0.9999/denv × chikv: *p* > 0.9999).

According to inflammatory infiltrate data, a more intense occurrence of inflammatory cells was observed in the YF and DF cases compared to the CF cases. In addition, lymphocytes and plasma cells were the cell specimens with the highest concentration.

## 4. Discussion

Arboviruses, such as YFV, DENV, and CHIKV, are responsible for diseases that pose risks to global health because they behave in the form of epidemics and epizootics epidemiologically, with seasonal outbreaks of greater or lesser impact on public health [4]. Although several studies have addressed the clinical manifestations and hepatic histological changes in YFV and DENV infections, little is known about the hepatic involvement in CHIKV infection or hepatic histopathological differences in fatal cases of YF, DF, and CF. Thus, the characterisation, quantification, and comparison of the patterns of histopathological changes in the liver identified in samples from patients affected by these infections helps to expand our knowledge about the process of viral pathogenesis and tissue damage caused by these viruses.

In our study of the semiquantitative histopathology of 70 liver samples from patients with YF, DF, and fatal CF, the quantification of histopathological changes showed abnormalities associated with cell swelling, macro- and microvesicular steatosis, lytic and coagulative necrosis, apoptosis, hyperplasia, hypertrophy of Kupffer cells, endothelial changes, and inflammatory infiltrate. Cellular swelling in the DF and CF cases showed homogeneous intensity, with no differences between the analysed acinus zones; however, for the CF and DF cases, the swelling was intense and moderate, respectively. An intensity variation was observed between the zones in the YF cases, in which the swelling was intense in Z1, and moderate in Z2 and Z3.

Although there are no data on swelling in the liver tissue of patients with CF, several studies have shown that YFV and DENV infections are associated with the development of hepatocyte swelling [53,54,55,56,57,58]. According to a study by Quaresma et al. [14], hepatocytes in Z1 and Z3 showed a greater intensity of this alteration. Cell swelling is attributed to changes in the mechanisms controlling water and ion concentrations due to direct viral action on the cell membrane or the immune response generated by the host [59,60]. Previous studies have reported several causes of the pathogenesis of these viruses that are inherent to the host and virus. The viral proteins nsP2 and nsP3 of the *alphavirus* CHIKV, and NS1, NS4A/B, and NS5, which belong to the *flaviviruses* YFV and DENV, play a role in the development of viral cytopathic effects. In addition to being associated with persistence in host cells, it promotes the attenuation of antiviral responses by inhibiting key factors in the immune response, resulting in viral dissemination in the host organism [13,47,48,49,50,51].

**Figure 9:**
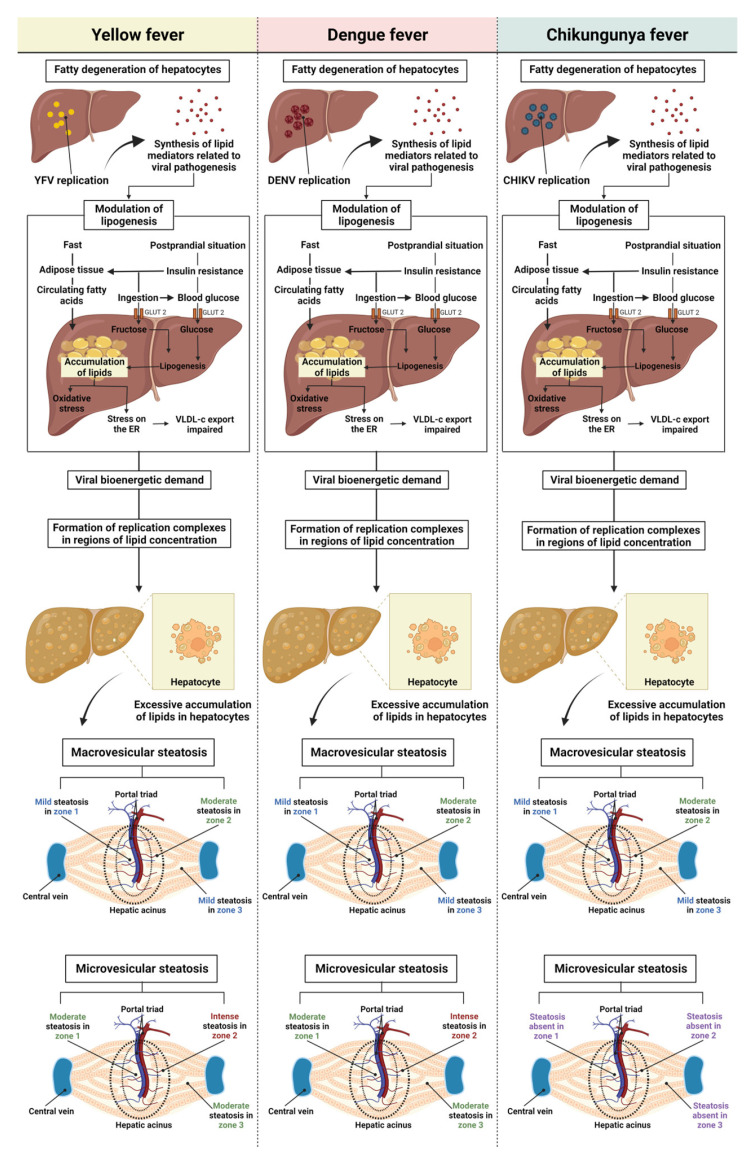
Steatosis in liver tissue of YFV, DENV, and CHIKV-infected patients. Hepatic steatosis is defined by an increase in lipids in hepatocytes. Microscopically, lipid droplets are observed in the liver parenchyma. The figure shows the mechanisms of macrovesicular and microvesicular steatosis. YFV, yellow fever virus; DENV, dengue virus; CHIKV, chikungunya virus.

Regarding macro- and microvesicular steatosis, the results showed no significant difference between the arboviruses, with uniform intensity in macrovesicular steatosis. However, a moderate difference in intensity was observed between the acinar zones, and the YF, DF, and CF cases showed a moderate increase in macrovesicular steatosis in Z2. In microvesicular steatosis, this alteration was not identified in the CF cases in any of the three evaluated areas, whereas similar intensity was observed in the YF and DF cases, with moderate microvesicular steatosis in Z1 and Z3 and intense microvesicular steatosis in Z2. Similar to macrovesicular steatosis, a preferential concentration of small lipid vesicles was observed in the cytoplasm of hepatocytes in the midzonal area. A predominance of microvesicular steatosis was also observed in the YF and DF cases, unlike the results for macrovesicular steatosis, in which there was no difference between the arboviruses studied (Figure 9).

In YF, macro- and microvesicular steatosis is a change commonly observed in infected patients, present in the three zones of the hepatic acinus but predominant in Z2 [57,61,62]. DF is associated with fatty liver degeneration [63,64]. Recently, Win et al. [65] reported extensive cell damage and microvesicular steatosis in human liver samples from patients who died of dengue haemorrhagic fever (DHF) [25]. Most studies have related YFV and DENV infections to the detection of microgoticular steatosis, whose presence is related to changes in lipid metabolism, one of the mechanisms involved in hepatomegaly and a clinical manifestation associated with hepatitis, observed in YF and DF [19,43,54].

Although data on hepatic fatty degeneration in CF are scarce, *flavivirus* and *alphavirus* infections have lipid metabolic implications that influence the immune response against infection and the maintenance of viral pathogenic mechanisms [47]. In addition, studies have reported that the synthesis of lipid mediators during viral infection is related to the signalling, control, and maintenance of the immune response and viral pathogenesis [66,67,68,69,70]. During infection, the virus can control the lipid metabolism of the host cell to meet the demands of the viral replication process, as the mobilisation and recruitment of lipid droplets is an essential mechanism in the bioenergetic demand for viral proliferation, which occurs through the formation of replication complexes in regions of high lipid concentration. Thus, the presence of steatosis in samples from patients infected with YFV, DENV, or CHIKV is expected, and may be linked to lipid metabolism [71,72,73,74,75,76,77,78,79].

**Figure 10:**
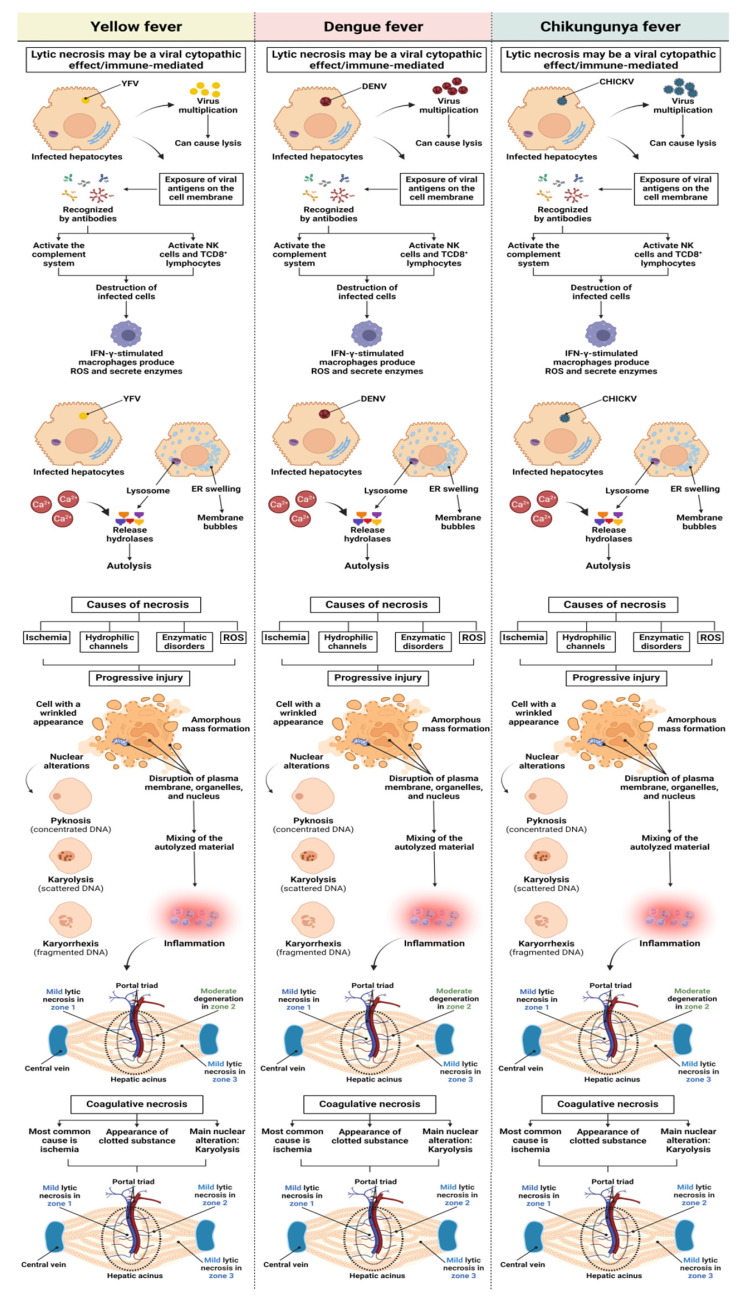
Necrosis in liver tissue of YFV, DENV, and CHIKV-infected patients. During the viral infection, infected hepatocytes undergo necrotic processes due to direct viral action on the infected cell and/or immunomodulation, activating cytotoxic cells such as NK and TC8^+^. YFV, yellow fever virus; DENV, dengue virus; CHIKV, chikungunya virus; NK, natural killer.

Regarding the necrotic processes analysed in the liver samples from the patients infected with YFV, DENV, and CHIKV, the degree of injury ranged from mild to moderate. There was no significant difference between arboviruses in lytic and coagulative necrosis, with an equivalent intensity of injury. However, for lytic necrosis, there was a preferential concentration of lesions in the midzonal area of the YF, DF, and CF samples, with a moderate degree of intensity. When evaluating coagulative necrosis, all cases in the three zones of hepatic acinus corresponded to a mild degree of this alteration, with no significant difference between the arboviruses or zones (Figure 10).

According to a study by Quaresma et al. [43], the intensity of steatotic hepatocytes coincides with that of apoptotic processes. In other studies, severe forms of DF were identified by the presence of hepatocellular necrosis, which is usually observed in Z1 and Z2 of the hepatic acinus [24,80]. In addition, several studies have associated the frequency of distinct histopathological findings in YF and DF with Z2 of the hepatic acinus; however, this pattern has also been observed in infections with other arboviruses [43,44,45,46,60,81,82,83,84]. In our study, Z2 had the highest concentration of damage, and the degree of injury varied from moderate to very intense, particularly in the case of YF, which showed the highest levels of harmful processes in the hepatic parenchyma.

According to Pan et al. [83], the development of injurious mechanisms in Z2 of the hepatic acinus is characteristic of the pathogenesis of *flavivirus* infections. This pattern of injury in the midzonal area is attributed to the intrinsic characteristics of hepatocytes, which have a lower presence of oxygen and nutrients than Z1 [40,64,68]. In a study by Olímpio et al. [62], lytic and coagulative necrosis were recurrent findings in the three hepatic zones of patients with YF; however, the highest frequency was observed in Z2. Triggering of these cell destruction processes may be related to the activation of a varied set of defence cells, generating a strong immune response characterised by a cytokine storm that causes tissue damage [35]. Hepatic histopathological findings from human cases of co-infection with DENV–CHIKV showed that patients had coagulative necrosis, especially around the hepatic vein [18,21,52]. In the animal models of CHIKV infection, infected mice presented foci of hepatocytic necrosis dispersed in the liver parenchyma without a specific zone on the third day after infection [85,86,87,88].

Regarding apoptosis, we observed significant differences between arboviruses; the CF cases showed an absence of this alteration in all analysed areas. In the DF cases, the results were homogeneous between zones, that is, apoptotic hepatocytes showed a homogeneous distribution in the liver parenchyma with moderate intensity in the evaluated areas. The YF cases were related to the highest values of apoptotic processes of the arboviruses studied, emphasising that the midzonal area presented a very intense lesion. The periportal and centrilobular areas of the YF samples showed intense and moderate apoptosis, respectively. From these data, it was possible to observe a clear predominance of alterations in the YF cases, particularly in Z2 (Figure 6).

**Figure 11:**
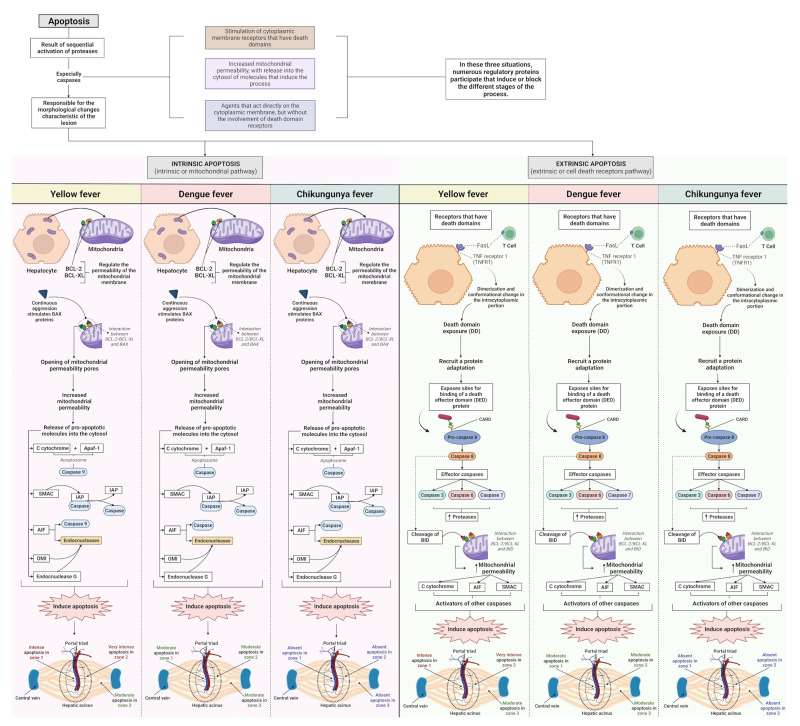
Apoptosis in liver tissue of YFV, DENV, and CHIKV-infected patients. In viral infection, the development of apoptotic processes is observed. The mechanisms of apoptosis induction involve intrinsic signals coming from mitochondrial alteration or by the activation of cell death receptors on the cell surface. YFV, yellow fever virus; DENV, dengue virus; CHIKV, chikungunya virus.

Apoptosis is a prominent histopathological feature of YFV infection in hepatocytes, Kupffer cells, and endothelial cells. In YF, liver injury mechanisms are complex and involve cytopathic effects inherent to viral replication and the development of the host immune response to neutralise viral replication by activating, producing, and releasing cytokines, chemokines, reactive oxygen species (ROS), and immune cells (Figure 11) [36,43,54,58,60,89,90]. This intense apoptotic event is also associated with the development of coagulopathy, as hepatocytes are the main producers of most circulating clotting factors; the loss of these factors via hepatocyte destruction or other mechanisms can result in an imbalance in the coagulation cascade, promoting haemorrhagic manifestation. The presence of apoptosis, lytic necrosis, swelling, and steatosis in hepatocytes is related to liver failure, which is a clinical manifestation observed in severe YF [91,92].

In the liver, two pathways of apoptosis were observed, extrinsic and intrinsic, which are dependent and independent of death receptors, respectively (Figure 11). Death receptors are the main mediators of apoptosis in the liver [93]. Enveloped viruses such as *flavivirus* and *alphavirus* are present in the outer layer of the host cell membrane derived from the endoplasmic reticulum (ER) and are mainly rich in phosphatidylserine (PS) and phosphatidylethanolamine (PE). PS and PE are relevant components for interactions with the host entry receptors and are important for tropism, pathogenicity, and viral infectivity processes. PS is mainly found in the outer membrane of apoptotic cells and is used as a “signal” for tissue macrophages. This mechanism is triggered by PS with viruses as binding molecules trigger “apoptotic mimicry” processes [94,95,96,97,98,99].

Data on hyperplasia showed a clear difference between the arboviruses in terms of alterations. The DF cases had a higher frequency of hyperplastic Kupffer cells, and an increase in the intensity of hyperplasia in the midzonal area of the YF, DF, and CF cases was also identified. Regarding hypertrophy, Z1 and Z2 in the YF, DF, and CF cases showed values corresponding to mild and moderate hypertrophy, respectively. In Z3, a difference between the arboviruses was observed; the CF cases showed moderate cellular hypertrophy, whereas the YF and DF cases showed mild hypertrophy. Unlike hyperplasia, the CF cases showed a higher frequency of moderate intensity in hypertrophy than the YF and DF cases. Regarding the evaluated zones, the midzonal area was more affected; however, in the case of CF, the midzonal and centrilobular areas were equally affected.

Kupffer cell hyperplasia and hypertrophy in YF are widely documented in previous studies [7,81,100]. Hyperplasia and Kupffer cell destruction were identified in most fatal cases of DF [25,64,101,102]. Liver injury observed in haemorrhagic fevers such as DF and YF revealed that hepatocytes and Kupffer cells might be the target cells for viral replication [53,103]. Viral antigens have been detected in Kupffer cells and hepatocytes from patients with DHF near the damaged areas, suggesting an association between viral replication and liver damage [104]. Viral antigens have also been detected in Kupffer cells of the liver samples from patients with fatal CF [22].

Three axes referring to hepatic endothelial alterations in the YF, DF, and CF cases were evaluated. In the comparative analysis, SEA was predominant in the YF and CF cases, with moderate intensity in the three different zones of the hepatic acinus. In relation to DF, the midzonal area of the cases was more affected, with a moderate degree of alteration, whereas in Z1 and Z3, the change was slight. In sinusoidal congestion, the CF cases did not show any change in any of the analysed areas; therefore, the prevalence of congestion was in the YF and DF cases, emphasising Z2 of the YF cases. In contrast to congestion, in sinusoidal dilatation, we observed that the cases of YF and DF did not present any alteration in any of the analysed zones. However, in the cases of CF, sinusoidal dilatation was moderate in all zones, that is, there was no specific zone of involvement.

Viral antigens have been detected in HSEC and Kupffer cells during arboviral infections [105,106,107,108,109]. In a study by Zellweger et al., a DENV-infected mouse model exhibited intense HSEC infection. Local HSEC injury may result in changes in portal microcirculation, resulting in secondary ischaemic injury and portal hypertension, which may be more pronounced in some patients [25]. Reversal of portal flow has also been demonstrated in *flavivirus* infections, which, together with circulatory dysfunction of the splanchnic vascular bed, may explain several features of liver dysfunction [109]. Sinusoidal congestion has been characterised in YF infections, with an intense circulation of macrophages and lymphocytes. Immune processes generate hepatocyte injury and necrosis of sinusoid capillaries owing to the release of cytokines, activation of the complement system, and endothelial dysfunction [61,110]. Two other studies related this change to fatal DF cases, indicating haemorrhagic congestion in hepatic sinusoids [101,102].

In a study by Sharp et al. [22], the liver samples from fatal CF cases showed the presence of the viral antigen in vessels, Kupffer cells, HSEC, and portal connective tissue; leukocytes were also found within the vascular channels of the liver. The most frequently identified histopathological features were chronic portal hepatitis (mild), steatosis (mild), rare hepatocyte necrosis/apoptosis, and sinusoidal congestion with leukocytosis. A study by Agarwal et al. [88] involving the hepatic histopathological analysis of mice infected with CHIKV demonstrated dilatation of the hepatic sinusoids in these animals as a result of the viral infection.

Of all the alterations identified in the study referring to PT, the main alterations analysed were moderate, with oedema being similar for the three arboviruses studied (Figure 11). In the alteration of the portal vein, the cases of DF were more intense. Congestion and alteration of the biliary canaliculus were mild and observed only in cases of YF and DF and absent in cases of CF. However, the alteration of the portal artery was mild for the three arboviruses studied. In a study by Duarte-neto et al., the microscopic liver analysis of YFV-infected individuals demonstrated the presence of oedema in the PT and the space of Disse. No patients had bile duct injuries.

Although the results obtained in the research related to histopathological alterations in DF were generally classified as mild to moderate, an explanation for these results could be the infectious serotype of the analysed samples. Given that the study did not aim at the molecular characterisation of the serotypes responsible for the infection, it is unknown whether the serotype of the samples examined was related to a different manifestation of DF, as the different clinical manifestations of the disease are also correlated with the DENV serotype. Patients infected with DENV-2 have a higher frequency of severe forms of the disease than those infected with other serotypes, such as persistent vomiting, epigastric pain, plasma leakage, and shock [111,112,113,114]. Among the serotypes, DENV-2 was most frequently isolated in cases of DHF and dengue shock syndrome, followed by DENV-3, DENV-1, and DENV-4 [16,115]. 

The acinar and portal inflammatory infiltrates had mild-to-moderate intensity, with lymphocytes and plasma cells identified as the main cell types. In the YF and DF cases, the concentration of acinar inflammatory infiltrates was more evident in Z2 than in the other zones of the hepatic acinus. Given the intense apoptotic process observed in the YF samples, the values related to inflammation were disproportionate to the intensity of liver injury. Our data corroborate previous findings in YF, in which the involvement of the liver parenchyma was disproportionate to the inflammatory findings. Furthermore, as previously mentioned, the pathogenic profile of YFV is correlated with the promotion of extensive apoptosis, a process linked to the non-induction of significant inflammatory response, thus explaining the scarcity of inflammatory infiltrates [35,36,43,116].

In the DF cases, as necrosis and apoptosis are generally associated with mild-to-moderate intensity, the limitation of inflammatory cells in the samples is predictable. Several studies show that inflammatory infiltrates in YFV and DENV infection cases mainly consist of lymphocytes [14,53,60,117]. Our results showed the same pattern; however, plasma cells also stood out among the analysed inflammatory infiltrates. Data from human studies collectively suggest an immunometabolic basis for symptomatic YFV infection. The anabolic demands of infection can exacerbate cellular stress and trigger ROS responses, cell death, and inflammation [118,119]. According to Chan et al., immunometabolic responses support symptomatic YF and possibly other *flavivirus* infections. Although CF is characterised by extensive inflammatory responses related to musculoskeletal injury, hepatic inflammatory involvement was not intense in our study. Studies analysing liver tissues from human cases and animal models of CHIKV infection have shown foci of mild inflammatory infiltrates composed of mixed inflammatory cells [52,88].

Infection promoted by flaviviruses and alphaviruses is based on the manipulation of host metabolism by controlling the survival of infected cells, thus affecting cell death pathways. Cell death pathways can be divided into two opposing processes, accidental cell death (ACD) and regulated cell death (RCD). Although ACD is a consequence of severe and rapid injury (osmotic forces, pH variations, and lytic viral replication), RCD is based on a regulated molecular machinery, implying modulation by pharmacological, genetic, or infectious components. Different cell death processes have different morphological characteristics, with three main types including apoptosis, autophagy-dependent cell death, and necrosis. Apoptosis is characterised by the formation of “apoptotic bodies,” autophagy-dependent cell death is defined by the extensive vacuolisation of the cytoplasm, and necrosis is mainly characterised by the rupture of the plasma membrane [120,121,122,123].

The activation of other described mechanisms of non-apoptotic RCD is directly linked to the viral load during infection. Necroptosis, which has morphological characteristics similar to necrosis, is an independent process involving caspases [124]. Pyroptosis is a highly inflammatory form of lytic RCD that is dependent on the formation of pores in the plasma membrane by members of the gasdermin (GSDM), a protein family which generally correlates with the activation of inflammatory caspases (CASP1, 4, or 5) [121,125]. These processes have been described in infections caused by intracellular pathogens and are related to the induction of antimicrobial responses and are involved in viral pathogenesis [126,127].

**Figure 12:**
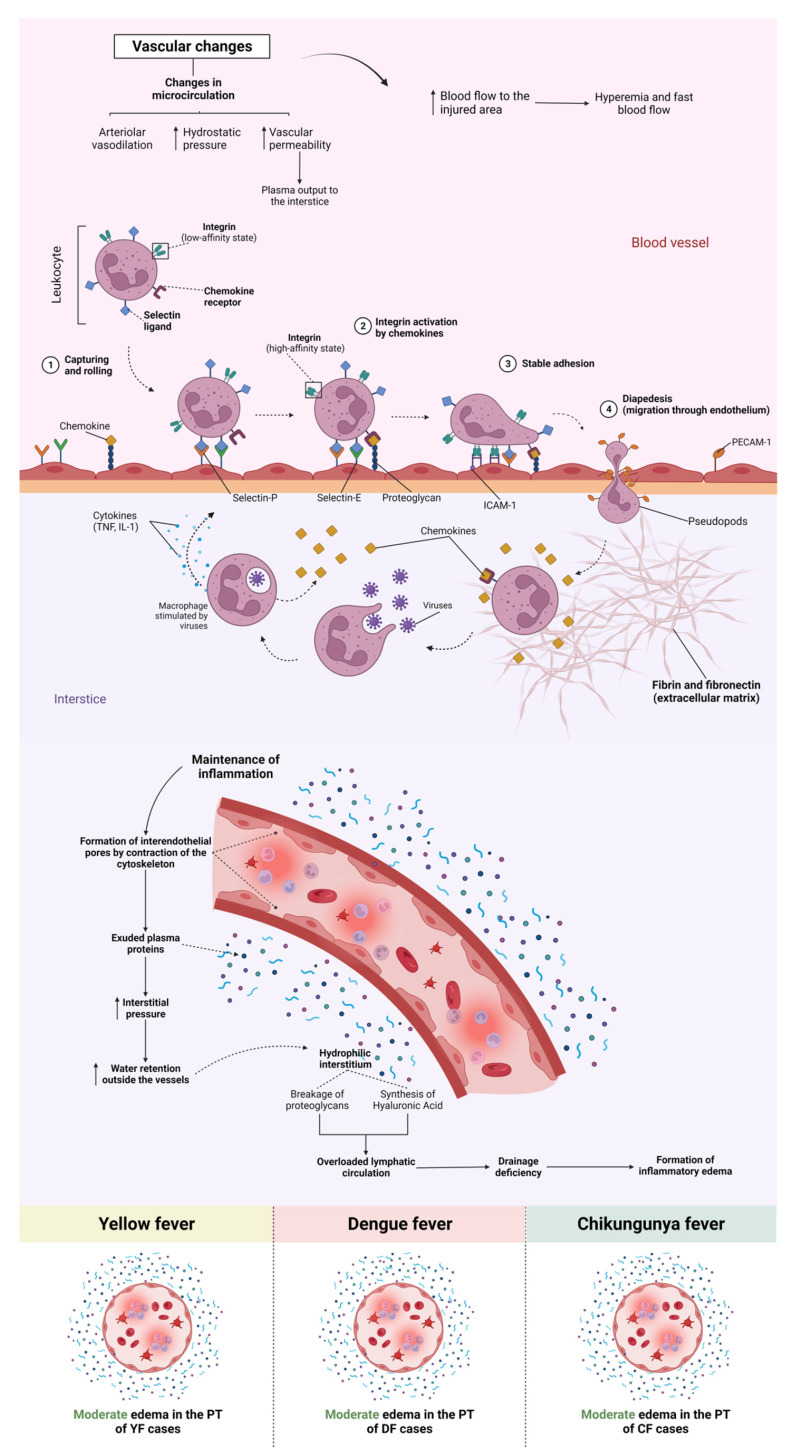
Vascular alteration in liver tissue of YFV, DENV, and CHIKV-infected patients. Changes in vascular microcirculation include arteriolar vasodilation. Smaller venules dilate, whereas larger venules undergo minor constriction, thereby increasing pressure in microcirculation, increasing vascular permeability, and plasma outflow into the interstitium. YFV, yellow fever virus; DENV, dengue virus; CHIKV, chikungunya virus.

Viral cytopathic mechanisms develop through the interaction between viral molecular components and cellular machinery. Flaviviruses and alphaviruses induce, activate, and inhibit several antiviral response mechanisms, including the development of different cell death processes, and influence the different morphological characteristics presented by cases of YFV, DENV, and CHIKV infection. Finally, our results indicated differences in the patterns of histopathological alterations in the human liver samples through fatal infections by YFV, DENV, and CHIKV related to hepatic acinus, portal tract, and inflammatory infiltrate, with intensity variation among the studied arboviruses and a predominance of lesions in certain areas of the liver parenchyma, resulting in tissue damage and liver dysfunction (Figure 12).

## 5. Conclusions

The cases of YFV, DENV, and CHIKV infections promoted different intensities of hepatic lesions in the analysed hepatic acinus zones (Z1, Z2, and Z3). There was a predominance of alterations in the hepatic acinus among the analysed areas, referring to the midzonal area (Z2) in YF, DF, and CF. Among the arboviruses studied, the hepatic involvement was more intense in the cases of YFV infection, characterising the classic hepatic profile of the disease.

In the semiquantitative evaluation, high-intensity cell swelling, microvesicular steatosis, and apoptosis were observed, classified as intense to very intense. Regarding the other histopathological changes evaluated in hepatic acinus, macrovesicular steatosis, lytic/coagulative necrosis, and Kupffer cell hyperplasia/hypertrophy ranged from mild to moderate.

The endothelial alterations in the hepatic acinus of the YF, DF, and CF cases varied from mild to moderate, with the YF and CF cases presenting with a higher frequency of moderate intensity. In the PT, a variation in intensity from mild to moderate was observed, emphasising oedema and portal vein alterations, with all arboviruses presenting a moderate degree of oedema. Patients with DF had relatively high values of portal vein alterations compared with those with YF and CF.

Infection with YFV, DENV, and CHIKV is accompanied by a mild-to-moderate inflammatory infiltrate consisting predominantly of lymphocytes and plasma cells, especially in cases of YF and DF, with a degree of intensity disproportionate to the involvement of the hepatic parenchyma. These data contribute substantially to the understanding of the pathogenesis of these arboviruses and the development of hepatotropic injury during infection, contributing to the improvement of diagnostic criteria for diseases, and expanding our knowledge of the pathogenesis of YFV, DENV, and CHIKV in human liver samples.

Future studies using IHC techniques, experimental models, and molecular biology techniques are necessary to define the cellular and molecular mechanisms involved in the tissue injury process associated with these arboviruses.

## Data Availability

The database used and/or analysed during the current study is not publicly accessible but can be available, upon reasonable request, from the corresponding authors.

## Supplementary Materials

The following supporting information can be downloaded at https://www.mdpi.com/article/10.3390/pathogens12050680/s1, Figure S1: Normal liver controls, showing absence of immunostaining and normal architecture and histology of hepatocytes and portal spaces. Magnification: 200× and 400×.
