# Supplementary figures and images for: Comparative Analysis of Human Hepatic Lesions in Dengue, Yellow Fever, and Chikungunya: Revisiting Histopathological Changes in the Light of Modern Knowledge of Cell Pathology
Source: Pathogens. 2023 May 4;12(5):680. doi: 10.3390/pathogens12050680 (PMC10222210; doi:10.3390/pathogens12050680)

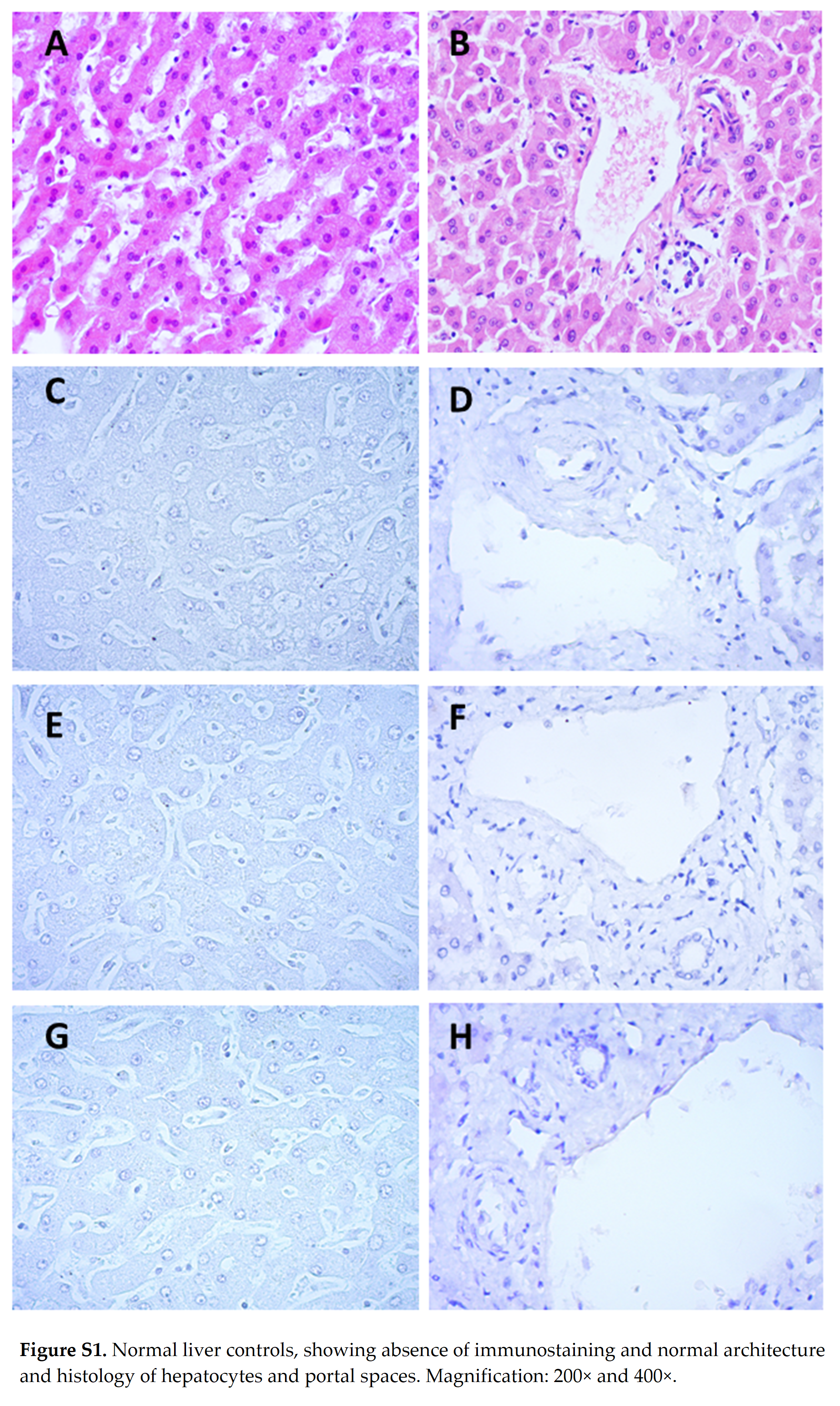

Supplement: Supplementary file 1 [file pathogens-12-00680-s001.zip › pathogens-2188326-supplementary.tif]
